# The Prefrontal Cortex Activity and Psychological Effects of Viewing Forest Landscapes in Autumn Season

**DOI:** 10.3390/ijerph120707235

**Published:** 2015-06-26

**Authors:** Dawou Joung, Geonwoo Kim, Yoonho Choi, HyoJin Lim, Soonjoo Park, Jong-Min Woo, Bum-Jin Park

**Affiliations:** 1 Department of Environment and Forest Resources, Chungnam National University, 99 Daehak-ro, Yuseong-gu, Daejeon 305-764, Korea; E-Mails: dawo.jeong@gmail.com (D.J.); bkim5020@gmail.com (G.K.); higure7@gmail.com (Y.C.); 2 Department of Applied Biology, Chungnam National University, 99 Daehak-ro, Yuseong-gu, Daejeon 305-764, Korea; E-Mail: hyojin328@gmail.com; 3 Department of Nursing, Eulji University, Daejeon 301-746, Korea; E-Mail: sjpark@eulji.ac.kr; 4 Department of Psychiatry, Seoul Paik Hospital, Inje University, School of Medicine, Seoul 100-032, Korea; E-Mail: jongmin.woo@gmail.com; 5 Stress Research Institute, Inje University, Seoul 100-032, Korea; E-Mail: jongmin.woo@gmail.com

**Keywords:** forest therapy, near-infrared spectroscopy, prefrontal cortex activity, hemoglobin concentration, semantic differential method, profile of mood states

## Abstract

Recently reported research indicate that forest environments have physiological and psychological relaxing effects compared to urban environments. However, some researchers claim that the stress of the subjects from being watched by others during measurements can affect the measurement result in urban experiments conducted in the center of a street. The present study was conducted to determine whether forest environments have physiological and psychological relaxing effects, using comparison of viewing a forest area with viewing an urban area from the roof of an urban building without being watched by others. Near-infrared spectroscopy (NIRS) measurement was performed on subjects while they viewed scenery for 15 min at each experimental site (urban and forest areas). Subjective assessments were performed after the NIRS measurement was complete. Total hemoglobin and oxyhemoglobin concentrations were significantly lower in the forest area than in the urban area. For semantic differential in subjective assessments, feelings of “comfortable”, “natural”, and “soothed” were significantly higher in the forest area than in the urban area, and for profile of mood states, negative emotions were significantly lower in the forest area than in the urban area. The results of physiological and psychological measurements show that viewing the forest enabled effective relaxation.

## 1. Introduction

In modern society, nature space is continuously decreasing owing to urbanization, and modern people live in a more complex and competitive environment compared to the past. In consequence, modern people are constantly exposed to stress. In an attempt to reduce the stress levels, many studies have been conducted to scientifically determine whether contact with forest has a physiological and psychological relaxing effect on human bodies [1,2,3,4,5,6,7,8,9,10]. At the beginning of these studies, the application of measurement technology to a field was limited; therefore, it was difficult to perform an experiment at a field. However, the rapid development of measurement technology and equipment accuracy has enabled accurate physiological measurements to be performed during field experiments [11].

Among the main physiological indicators used in a field experiment, autonomic nervous system activity indicators include blood pressure [1,2,3,7], pulse rate [1,2,3,4,6,7], and heart rate variability [1,2,3,4,5,6], and endocrine system activity indicators include salivary cortisol concentration [2,3,4,6,7,8,4,6]. An immune system activity indicator is natural killer cell activity [9,10]. Time-resolved spectroscopy (TRS) [8] is currently used as a central nervous system activity indicator.

However, stimulus from various external factors cannot be controlled in field experiments. External factors can be physical environment such as temperature, humidity and wind speed or the awareness of the external environment of the subjects. And subject awareness of the gaze of others during measurement can affect the measurement result in urban experiments, particularly in the center of a street. Accordingly, the present study was conducted in a forest and on the roof of a building, away from the view of others, to minimize external stimuli.

It has been recently proposed that the effect of forest therapy can be scientifically demonstrated using near-infrared spectroscopy (NIRS), considering reports that the prefrontal cortex activity, following a stimulus causing stress is closely associated with a change in autonomic nervous functions or state of mind and body [12,13,14]. The present study was conducted to evaluate the physiological and psychological effects of viewing forest scenery on prefrontal cerebral activity using portable NIRS and subjective assessments.

## 2. Methods

The study was conducted in a forest located at Dowon-ri, Toseong-myun, Goseong-gun, Gangwon-do, Korea. An urban area where buildings are concentrated in Yuseong-gu, Daejeon Metropolitan City, Korea, was selected as a contrasting site. The urban experiment of the study was conducted on the rooftop of a four-story building. The subjects were directed to view the urban scenery on the roof of a building. Before the experiment, it had been notified that if the subject feels any discomfort such as “fear of heights” or “physical disorder” he or she can terminate the experiment at their own will.

Eight Korean university students (age range 22.0 ± 2.2 years) participated in this study. The subjects were physically and mentally healthy adults. This study was performed according to the regulations of the Bioethics Committee of Chungnam National University in Korea.

NIRS [15] was used to evaluate the physiological reaction. NIRS measured human central nervous system activity indicators using a portable near-infrared tissue oxygenation monitor (PocketNIRS Duo, Dynasense, Shizuoka, Japan) [16,17]. Moreover, NIRS measured changes in the hemoglobin concentration in the prefrontal cortex via the modified Beer–Lambert (MBL) method [18] from a change in the intensity of radiation of each of three wavelengths: 735, 810, and 850 mm. In this study, a change was measured in the total hemoglobin (total Hb) and oxyhemoglobin (oxy-Hb) concentrations. The NIRS data were obtained by performing measurement for a total of 15 min per experiment area and were collected at an interval of 1 s.

Psychological assessments, including the semantic differential (SD) method and a profile of mood state (POMS) were applied to evaluate psychological reactions. The SD method [19] is widely used to evaluate scenery, a factor difficult to quantify owing to subjective differences. The study evaluated feelings on a 13-point scale using adjectives, such as “comfortable–uncomfortable”, “natural–artificial”, and “soothed–stimulating”. This study used a shortened version of POMS, which is a method of evaluating the emotions of respondents in items, such as “tension and anxiety”, “depression”, “anger and hostility”, “vigor”, “fatigue”, “confusion”, and “total mood disturbance” using 30 questions [20,21,22].

**Figure 1:**
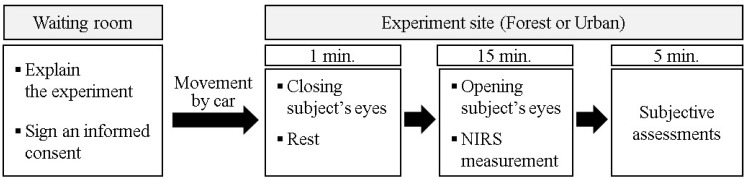
Experimental design.

Figure 1 shows an experimental design of this research. All subjects were informed of the contents of experiments before starting the experiments, understood the experimental method, and then participated in the experiment; the subjects agreed to all the contents of the experiment.

The experiment comprised 2 sessions. In the first session, forest scenery was viewed, and in the second session, urban scenery was viewed. The experiments in this study were designed with “Single-Group Crossover Design” [23].

To minimize the quantity of motion, the subjects were moved by car from waiting room to experiment sites. The subject walked 10 m on foot after getting out of car. The subjects were asked to sit on chair and close their eyes immediately and 1 min was given for subjects’ settlement. After 1 min settlement, the subjects were asked to open eyes and NIRS were measured for 15 min. The subjective assessments were evaluated after NIRS measurement.

Eight samples were collected and analyzed. For the NIRS data, a paired t-test was used to compare the forest and urban area results. Only 7 samples were used, owing to an error in measurement of 1 subject. The subjective assessments were performed with a Wilcoxon signed-rank test. Statistical analyses were performed with Excel 2010 (Microsoft Inc., Redmond, WA, USA) and SPSS 21.0 (SPSS Inc., Chicago, IL, USA). For all cases, *p* < 0.05 was considered statistically significant. 

## 3. Results and Discussion

**Figure 2:**
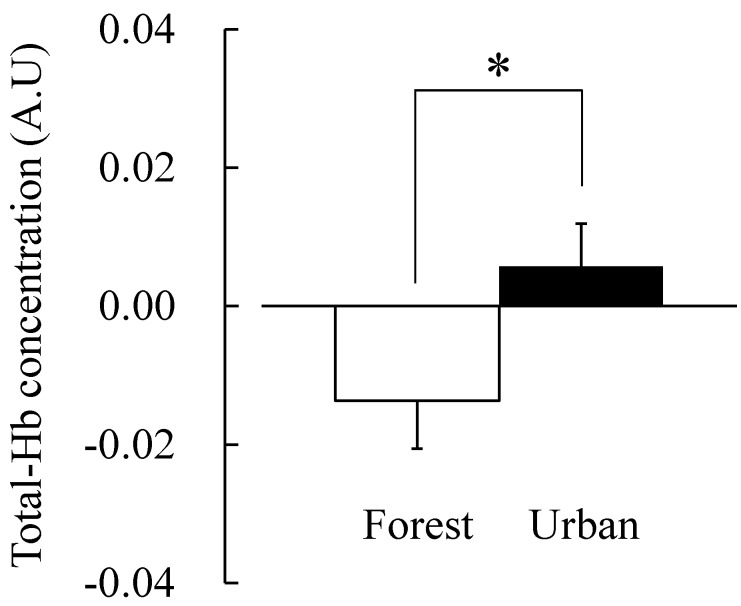
Comparison of the total-Hb concentration of subjects viewing forest and urban areas. N = 7, mean ± SE, * *p* < 0.05, determined by paired *t*-test.

**Figure 3:**
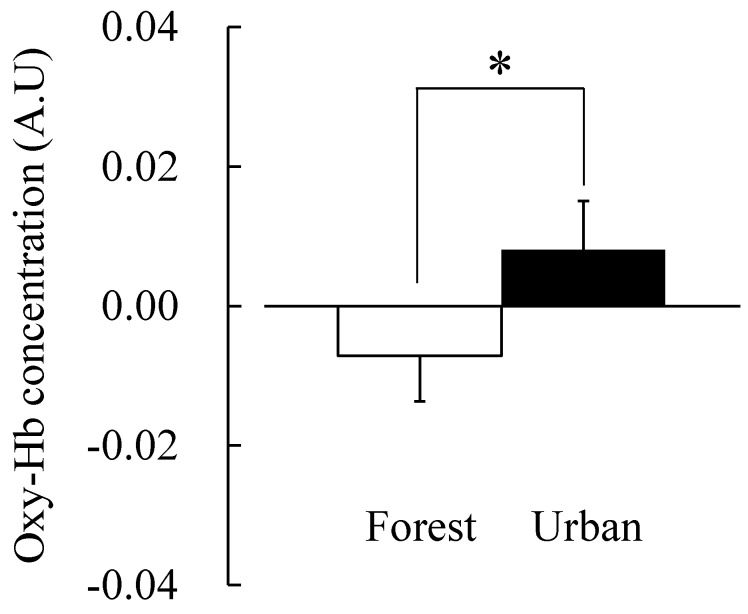
Comparison of the oxy-Hb concentration of subjects viewing forest and urban areas. N = 7, mean ± SE, * *p* < 0.05, determined by paired *t*-test.

The change in total Hb concentration was significantly lower when forest scenery was viewed (−0.014 ± 0.007 A.U.) than when urban scenery was viewed (0.006 ± 0.006 A.U.; *p* < 0.05; Figure 2; Table 1). The change in oxy-Hb concentration was also significantly lower in the forest area (−0.007 ± 0.007 A.U.) than in the urban area (0.008 ± 0.007 A.U.; *p* < 0.05; Figure 3; Table 1). A lower concentration of total Hb and oxy-Hb indicates that the quantity of oxygen transmitted to the prefrontal cortex tissue is small. In other words, the prefrontal cortex activity in a forest area is more stabilized than that in an urban area. This result is consistent with that of a previous study [8,17]; Park *et al.* (2007) and Ikei *et al.* (2013) showed that low Hb concentration means the relaxation of brain function [8,17], and Park *et al.* (2007) reported that the absolute value of cerebral activity in the prefrontal area of the forest area was significantly lower than in the urban area [8].

**Table 1:** Summary of the comparison of hemoglobin concentration between the forest and urban areas.

	Total Hb	Oxy-Hb
Forest area	−0.014 ± 0.007	−0.007 ± 0.007
Urban area	0.006 ± 0.006	0.008 ± 0.007
*p*-value	0.023 *	0.014 *

N = 7, mean ± SE, * *p* < 0.05.

**Figure 4:**
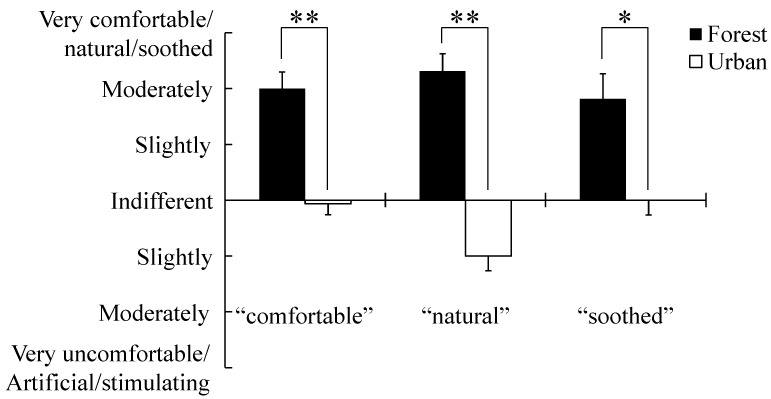
Comparison of subjective scoring for “comfortable”, “natural”, and “soothed” feeling between forest and urban areas. N = 8, mean ± SE, * *p* < 0.05; ** *p* < 0.01, determined by Wilcoxon signed-rank test.

**Figure 5:**
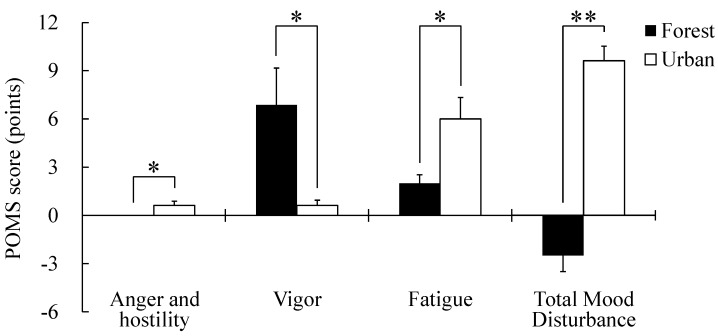
Comparison of subjective scoring for “anger and hostility”, “vigor”, “fatigue”, and “total mood disturbance” by POMS between forest and urban areas. N = 8, mean ± SE, * *p* < 0.05; ** *p* < 0.01, determined by Wilcoxon signed-rank test.

Comparison of the SD scores showed that the feelings of “comfortable”, “natural”, and “soothed” were all significantly higher in the forest area than in the urban area (*p* < 0.05; Figure 4; Table 2). Figure 5 and Table 3 show comparison of the POMS scores in the two areas. “Anger and hostility”, “fatigue”, and “total mood disturbance” were significantly lower in the forest area than in the urban area (*p* < 0.05). Furthermore, “vigor” was significantly higher in the forest area than in the urban area (*p* < 0.05). However, “tension and anxiety”, “depression”, and “confusion” were not significantly different between the two areas. 

This result was partly consistent with those of previous studies, indicating that the forest area has more positive effects than the urban area [7,8,24,25].

**Table 2:** Summary of the comparison of SD method between the forest and urban areas.

	Comfortable	Natural	Soothed
Forest area	4.00 ± 0.60	4.63 ± 0.63	3.63 ± 0.91
Urban area	0.13 ± 0.40	−2.00 ± 0.53	0.00 ± 0.53
*p*-value	0.006 **	0.006 **	0.010 *

N = 8, mean ± SE, * *p* < 0.05, ** *p* < 0.01

**Table 3:** Summary of the comparison of POMS between the forest and urban areas.

	T-A	D	V	F	C	A-H	TMD
Forest area	0.63 ± 0.38	0.13 ± 0.13	6.88 ± 2.29	2.00 ± 0.53	1.63 ± 0.65	0.00 ± 0.00	−2.50 ± 1.49
Urban area	2.25 ± 0.73	0.25 ± 0.25	0.63 ± 0.32	6.00 ± 1.34	1.13 ± 0.30	0.63 ± 0.26	9.63 ± 0.91
*p*-value	0.062	0.327	0.014 *	0.021 *	0.248	0.029 *	0.006 **

T-A: anger-hostility; D: depression; V: vigor; F: fatigue; C: confusion; A-H: anger-hostility; TMD: total mood disturbance. N = 8, mean ± SE, * *p* < 0.05, ** *p* < 0.01.

Meanwhile, this study has some limitation. First, There were only 8 subjects in the study. Considering the small quantity of subjects, we defined the subjects’ age in their 20’s. Therefore, it is difficult to estimate in other age ranges. To generalize the result of the study, there needs to be evidence-based further research on a larger sample size including various age ranges. Second, NIRS measurement instrument used for this study can show only the changing value from the beginning of measurement and can’t indicate the absolute value. The zero point is the value at the beginning of each measurement. Therefore, this study directly compares the difference of NIRS data changes between when viewing of forest and urban areas. Third, only NIRS was used to evaluate physiological parameters. However, the application of NIRS to demonstrate the effect of forest therapy is supported by recent findings stating that the measurement of prefrontal cortex activity via NIRS can be applied to the objective assessment of stress condition and relaxation effects [13,14].

## 4. Conclusions

This study was conducted to evaluate the physiological and psychological effects of viewing scenery in a forest area. The prefrontal cortex activity in a forest area was more stabilized than in an urban area, and the forest area produced more relaxing effects than did the urban area. Considering that the urban part of the study was conducted on the roof of an urban building out of the view of others, these results suggest that a forest area produces more physiological and psychological relaxing effects than an urban area even when others are not watching.
